# FBAT DAP-G: fine-mapping of genetic associations in family-based studies using the Deterministic Approximation of Posteriors algorithm

**DOI:** 10.1093/bioadv/vbaf233

**Published:** 2025-10-01

**Authors:** Julian Hecker, Dmitry Prokopenko, Sanghun Lee, Sharon M Lutz, Rudolph E Tanzi, Christoph Lange

**Affiliations:** Channing Division of Network Medicine, Brigham and Women’s Hospital and Harvard Medical School, Boston, MA 02115, United States; Genetics and Aging Research Unit and McCance Center for Brain Health, Department of Neurology, Massachusetts General Hospital and Harvard Medical School, Boston, MA 02129, United States; Department of Medical Consilience, Division of Medicine, Graduate School, Dankook University, Yongin-si, Gyeonggi-do 16890, South Korea; Department of Biostatistics, Harvard T.H. Chan School of Public Health, Boston, MA 02115, United States; Department of Biostatistics, Harvard T.H. Chan School of Public Health, Boston, MA 02115, United States; Department of Population Medicine, Harvard Pilgrim Health Care Institute and Harvard Medical School, Boston, MA 02215, United States; Genetics and Aging Research Unit and McCance Center for Brain Health, Department of Neurology, Massachusetts General Hospital and Harvard Medical School, Boston, MA 02129, United States; Channing Division of Network Medicine, Brigham and Women’s Hospital and Harvard Medical School, Boston, MA 02115, United States; Department of Biostatistics, Harvard T.H. Chan School of Public Health, Boston, MA 02115, United States

## Abstract

**Motivation:**

Family-based study designs enable genetic association analyses that are robust against population stratification and admixture. The Family-Based Association Test (FBAT) framework extended and generalized the Transmission Disequilibrium Test concept for trios (affected offspring and their parents) to general pedigrees, arbitrary phenotypes, and multi-variant analyses. Following the successful identification of disease susceptibility loci in classical family studies, FBAT approaches can be a valuable tool in modern large-scale biobanks that contain significant proportions of related individuals, often with heterogeneous genetic ancestries. However, recent methods for established downstream analyses, such as statistical fine-mapping, are primarily tailored towards genetic association results obtained from regression models in unrelated individuals, and suitable methodologies for FBAT results have not been established in the literature. Here, we introduce and implement a framework for statistical fine-mapping in FBAT-based family-based association studies.

**Results:**

Our approach is based on the established Deterministic Approximation of Posteriors algorithm and utilizes the association *z*-scores obtained from FBAT. We illustrate the method in simulation studies and an application to the Apolipoprotein E locus in a family-based association study of Alzheimer’s Disease.

**Availability and implementation:**

The fine-mapping approach FBAT DAP-G is implemented in the FBAT package https://github.com/FBATsw/FBAT/. The original code of DAP-G is available at https://github.com/xqwen/dap.

## 1 Introduction

Family-based study designs enable genetic association testing that is robust against population stratification and admixture (Laird and Lange 2006). The most popular example is the Transmission Disequilibrium Test (TDT), which assesses deviations from Mendelian transmission in affected offspring and their parents (Spielman *et al.* 1993). By utilizing the seminal work of Rabinowitz and Laird on sufficient statistics in the family-based context (Rabinowitz and Laird 2000), the Family-Based Association Test (FBAT) approach constructs conditional score-based tests that maintain the robustness of the TDT in more general scenarios, including nuclear families with missing parental genetic data and multiple offspring, arbitrary phenotypes, and multiple genetic variants (Laird and Lange 2006).

Similar to case-control designs or the general analysis of unrelated individuals in genome-wide association studies (GWAS), significant genetic associations in genome-wide FBAT analyses do not directly point to causal genetic variants due to indirect associations caused by linkage disequilibrium (LD). In particular, FBAT tests the null hypothesis of no linkage and no association, implying that significant genetic associations can arise if the causal variant is in LD with the tested variant, but they do not coincide. This is in agreement with the observation of GWAS peaks in candidate loci with multiple significantly associated genetic variants, which led to the development of so-called statistical fine-mapping approaches. Statistical fine-mapping aims to identify the causal genetic variants influencing the complex trait underlying a corresponding GWAS locus, assuming that at least one causal variant exists. In the following, we adopt this assumption.

Following early approaches using heuristics or penalized regression models, recent fine-mapping methods are commonly based on Bayesian models. These Bayesian models aim to infer so-called posterior inclusion probabilities (PIPs), providing a measure of evidence for the causal effect of a genetic variant. Initially starting with the assumption that at most one causal genetic variant is located in the locus of consideration, recent approaches have been proposed that can identify multiple causal genetic variants by using sophisticated techniques to circumvent the high computational complexity of these models. Examples of Bayesian statistical fine-mapping approaches include FINEMAP (Benner *et al.* 2016), CAVIAR (Hormozdiari *et al.* 2014), and SuSiE (Wang *et al.* 2020, Zou *et al.* 2022).

Another particular example of a Bayesian fine-mapping approach that can model the existence of multiple causal variants is the Deterministic Approximation of Posteriors (DAP) method (Wen *et al.* 2016), which has been extended to be applicable to association summary statistics (DAP-G) (Lee *et al.* 2018). In particular, DAP-G can fine-map genetic risk loci based on association *z*-scores and an estimated correlation matrix that captures the dependencies between the genetic variants due to LD. Furthermore, DAP-G allows the incorporation of variant-specific prior information based on functional annotations or other external data. As we will illustrate, these features make the DAP-G approach appealing for the statistical fine-mapping of FBAT results. Here, we introduce an adaptation of the DAP-G fine-mapping method for family-based studies, which is readily available in the FBAT software. We demonstrate its application in simulation studies and by analyzing the Apolipoprotein E (APOE) locus in a family-based association study of Alzheimer’s Disease (AD).

## 2 Approach

FBAT association testing is based on score tests that condition on the observed phenotypes and the sufficient statistic for the parental genotype distribution within each nuclear family. Due to the nature of score tests, FBAT does not provide estimated genetic effect sizes; instead, it directly computes association z-scores. While recent fine-mapping methods for GWAS with unrelated samples mainly aim to recover or approximate full-data model computations based on summary statistics (effect estimates, estimated standard errors, etc.) (Zou *et al.* 2022), fine-mapping of FBAT association statistics demands an approach that models z-scores within a given locus directly. In particular, we assume that the vector of z-scores for the genetic region of interest can be approximately modeled by Chen *et al.* (2015).


Z∼N(Rβ,R),

where R denotes a matrix that describes the correlation between the z-scores under the null hypothesis. Further, we assume that the non-zero entries of the sparse vector β correspond to a binary configuration vector γ (drawn according to a specified prior distribution), and non-zero entries are drawn independently from a mean-zero normal distribution with fixed variance. This model entails the same Bayes Factor computations as those considered in the DAP-G fine-mapping algorithm and its corresponding implementation (Lee *et al.* 2018). Here, we thus introduce FBAT DAP-G, which implements the established DAP-G fine-mapping technique in the FBAT framework. We note that FBAT can compute the input Z for different genetic models (additive, recessive, and dominant), and the corresponding R can be approximately estimated from in-sample offspring genetic data using the respective genetic model coding. In the case of a recessive or dominant model, there are two distinct *z*-scores for each genetic variant available (one for each allele). Therefore, in the following, we refer to genetic factors or genetic tests in the region of interest, instead of genetic variants.

DAP-G presents a Deterministic Approximation of Posteriors (DAP) that can efficiently compute PIPs for each genetic test within a locus. This is possible since DAP-G utilizes the observation that most of the posterior probability mass in the space of all possible combinations is concentrated in models with only a few genetic factors included. DAP-G adaptively selects a small subset of candidate factors and applies a combinatorial approximation to estimate the posterior probability mass from the unexplored model space. We refer to the DAP (Wen *et al.* 2016) and DAP-G (Lee *et al.* 2018) publications for further details. We also note that DAP-G was studied in extensive simulation studies and compared to other approaches recently (Zou *et al.* 2022). DAP-G requires the specification of hyperparameters and prior probabilities. The hyperparameters include the maximum size of models and within-correlation thresholds for signal clusters, but most importantly, the threshold parameter, denoted here by λ, to control the stopping rule of the approximation algorithm. In particular, λ specifies a threshold on the log-scale to add candidate factors to the model space. A higher value of λ explores the model space in more detail. For all hyperparameters, we applied the default values as described in the DAP-G implementation, but assessed the impact of different values for λ in the simulations. Furthermore, DAP-G specifies the prior probability of a non-zero genetic effect for a factor by default as the reciprocal of the number of factors in the region under consideration. However, DAP-G can also incorporate external information to specify factor-specific prior probabilities, thereby reflecting additional prior evidence. This enables the incorporation of another feature of FBAT-based family-based association studies into the fine-mapping procedure: The FBAT approach allows the construction of independent screening statistics based on sufficient statistics, which can inform downstream FBAT analyses (Laird and Lange 2006). This information can be used to design factor-specific prior probabilities for each test in the fine-mapping region.

## 3 Results

### 3.1 Simulations

We performed simulation studies to validate and illustrate our implementation of the FBAT DAP-G fine-mapping framework.

We considered two different study designs: (i) trios (genetic data for offspring and both parents) and (ii) siblings with missing parental genetic data (genetic data for two full siblings). Furthermore, we incorporated additive and recessive genetic models, and scenarios with 1 or 2 causal effects. We considered three different choices for the fine-tuning parameter λ, given by λ=2.0, 1.3, 0.097, whereas λ=2.0 is considered the default option in the DAP-G implementation. Further details are described in the Supplementary Materials, available as supplementary data at *Bioinformatics Advances* online. As a direct comparison with an alternative method, we applied the CAVIAR approach (Hormozdiari *et al.* 2014) to the vector of *z*-scores and the corresponding correlation matrix, as computed by FBAT. CAVIAR performs more exhaustive Bayesian computations (maximum number of causal variants set to 2 in our investigations) and imposes a significantly higher computational burden (Zou *et al.* 2022).

**Figure 1.**
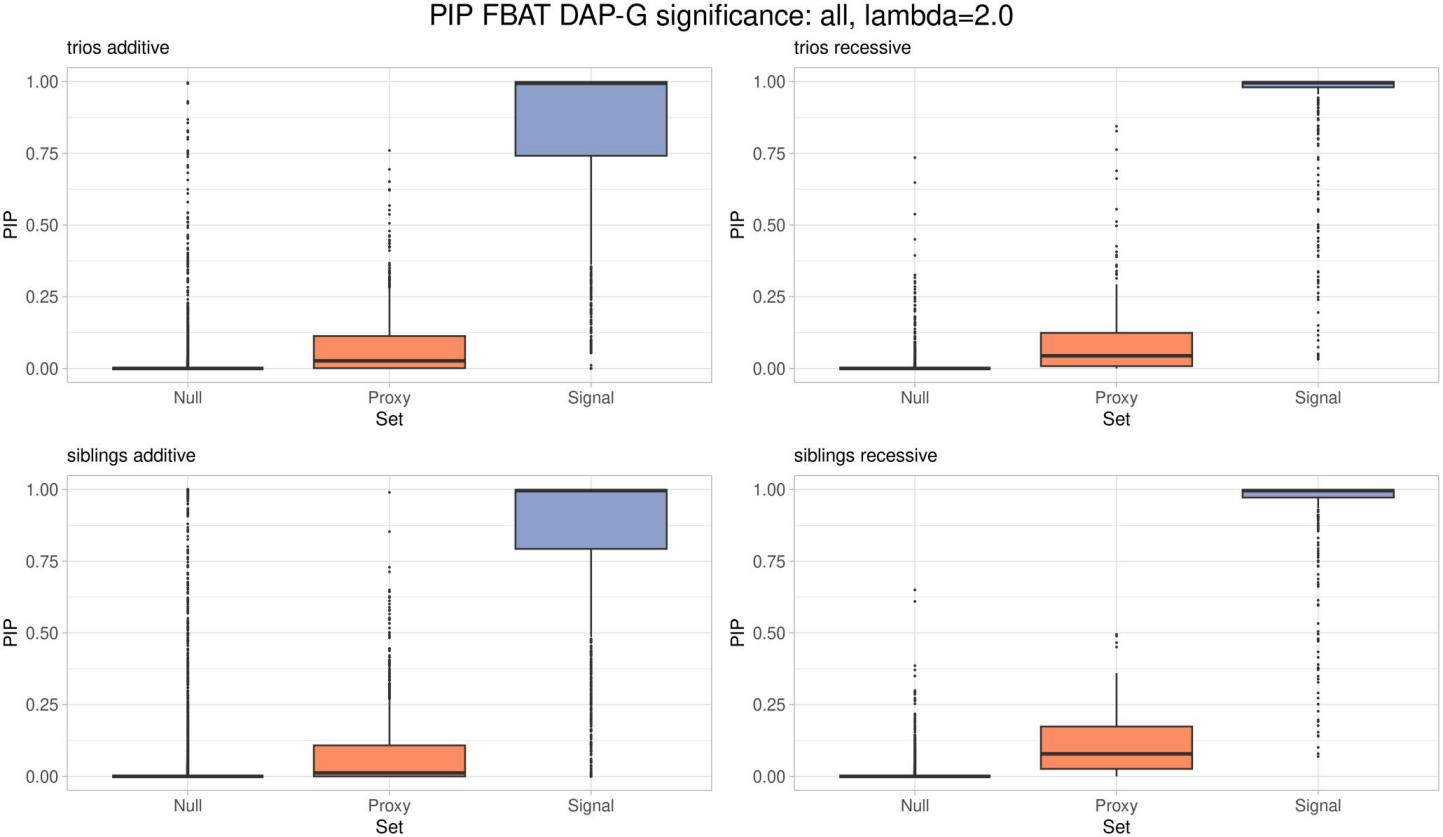
Comparison of posterior inclusion probability distributions for significant loci. FBAT DAP-G posterior inclusion probabilities (PIPs) across all simulated replicates in which all true causal genetic effects achieved genome-wide significance (*P* < 5×10^−8^). Results are shown stratified by study design and genetic model, leading to three different “Sets”. “Signal” denotes genetic association tests with true effect, “Proxy” are genetic association tests in strong correlation ($r^{2}\geq 0.8$) with causal genetic effects (LD in the case of an additive model). “Null” denotes genetic association tests with no effect and not in correlation with signals. FBAT DAP-G parameter λ=2.0. PIP: posterior inclusion probability.

Figure 1 visualizes the distribution of FBAT DAP-G PIPs across all simulated replicates in which all true causal genetic effects achieved genome-wide significance (*P* < 5 × 10^−8^) for genetic association tests that (i.) possess true effects, (ii) are close correlation proxies to causal effects ($r^{2}\geq 0.8$), and (iii) have no genetic effect on the outcome and are not in strong correlation with any causal effect, stratified by the genetic model and study design (using default parameter option λ=2.0). Figures 2 and 3, available as supplementary data at *Bioinformatics Advances* online, visualize the same data for FBAT DAP-G with λ=1.3 and CAVIAR, respectively. Figures 4–6, available as supplementary data at *Bioinformatics Advances* online, correspond to the information displayed in Figs. 1–3 (Figs 2 and 3 available as supplementary data at *Bioinformatics Advances* online), but are now restricted to scenarios in which no causal genetic effect achieved genome-wide significance.

The figures suggest that study design and mode of inheritance have minimal impact on the results. Negligible PIP mass is assigned to genetic factors that are neither causal nor strongly correlated with causal variants. As expected, the box plots show that causal factors tend to receive higher PIPs when their marginal association *P*-values are highly significant. However, for causal variants that do not reach genome-wide significance, the PIP mass is more diffuse, reflecting statistical uncertainty.

Furthermore, Fig. 7, available as supplementary data at *Bioinformatics Advances* online, reports the calibration of PIPs across all scenarios, for FBAT DAP-G and CAVIAR. Figure 8, available as supplementary data at *Bioinformatics Advances* online, illustrates the relationship between the absolute value of association *z*-scores and the corresponding PIP for the causal genetic effects in scenarios with exactly one causal effect for FBAT DAP-G (λ=2.0). Finally, we also included a precision-recall curve plot for all methods in Fig. 9, available as supplementary data at *Bioinformatics Advances* online. This figure demonstrates the strong ability of FBAT DAP-G and, especially, the underlying DAP-G algorithm to classify genetic factors with both high precision and high recall while imposing a strongly reduced computational burden.

Overall, the results, summarized by these figures, show that (i) FBAT DAP-G fine-mapping can identify underlying genetic signals across different study designs and the assumed modes of inheritance, (ii) the overall ability to fine-map is related to the strength of the genetic signal, (iii) the FBAT DAP-G PIPs are well calibrated, (iv) the performance of FBAT DAP-G in the simulated scenarios is not strongly impacted by the selection of λ, and (v) CAVIAR performs comparably across the scenarios, but we emphasize the significantly larger computational burden because it exhaustively evaluates more SNP configurations, as previously described (Zou *et al.* 2022). We investigated the last point in more detail by performing a runtime comparison between DAP-G and CAVIAR, confirming this observation (Tables 2 and 3, available as supplementary data at *Bioinformatics Advances* online).

### 3.2 Application to a family-based association study of AD

We applied the FBAT DAP-G fine-mapping approach to a family-based association study of AD, focusing on the well-established APOE risk locus (±500 kb from the known rs429358 risk variant). Further details can be found in the Supplementary Materials. The FBAT DAP-G fine-mapping analysis, using an additive genetic model, identified two signal clusters with PIP > 40% (Table 1, available as supplementary data at *Bioinformatics Advances* online). The first signal cluster achieved a combined PIP of 99.7% and contained the four genetic variants rs429358, rs12721051, rs4420638, and rs56131196. Here, rs429358 achieved the highest variant-specific PIP of 36.2%, the single variant FBAT test for this SNP yielded an association *P*-value of (*P* = 4.4 × 10^−16^) (133 informative families). The SNP rs429358 is an established risk variant for Alzheimer’s disease (AD) and one of two SNPs that define the epsilon alleles at the APOE locus. The other of these two SNPs, rs7412, is a low-frequency variant and was not identified by the fine-mapping approach as part of a signal cluster. The corresponding single-variant FBAT yielded an association *P*-value of *P* = 6.2×10^−5^ (29 informative families), representing only weak evidence due to the limited sample size and potentially hindering detection in the fine-mapping analysis. The second signal cluster comprised 18 genetic variants and achieved a combined PIP of 42.4%, indicating weak evidence. The strongest marginal association within this cluster was observed for rs11666285 (*P* = 0.000233, 74 informative families). This variant was previously reported to be associated with a family history of AD (see Supplementary Materials, available as supplementary data at *Bioinformatics Advances* online). In Fig. 10, available as supplementary data at *Bioinformatics Advances* online, we visualized the region plot, PIP information, and LD heatmap for the identified signal clusters. Furthermore, we repeated the analysis using a recessive genetic model. The results of this analysis did not reveal any additional insights (Fig. 11, available as supplementary data at *Bioinformatics Advances* online) and suggest that the additive genetic model provides a more accurate description of the data. Overall, the results illustrate a successful application of FBAT DAP-G to this established AD risk locus.

## 4 Discussion

We introduced an adaptation of the DAP-G statistical fine-mapping approach for FBAT-based association studies and implemented it in the FBAT software. We validated the method in simulations and applied it to the APOE locus in an family-based AD dataset, where we identified two signal clusters with a posterior inclusion probability greater than 40%. Providing an efficient implementation of the fine-mapping downstream analysis represents a crucial tool for family-based approaches, as interest in these analyses is growing in the era of large-scale biobanks.

## Supplementary Material

vbaf233_Supplementary_Data

## Data Availability

The fine-mapping approach FBAT DAP-G is implemented in the FBAT package https://github.com/FBATsw/FBAT/. The original code of DAP-G is available at https://github.com/xqwen/dap. The National Institute of Mental Health AD dataset is available upon reasonable request.
