# Supplementary material for: FBAT DAP-G: fine-mapping of genetic associations in family-based studies using the Deterministic Approximation of Posteriors algorithm
Source: Bioinform Adv. 2025 Oct 1;5(1):vbaf233. doi: 10.1093/bioadv/vbaf233 (PMC12509871; doi:10.1093/bioadv/vbaf233)
Supplement: vbaf233_Supplementary_Data [file vbaf233_supplementary_data.zip › supplementary_materials_bioinf_advances.pdf]

Supplementary Materials

Appendix

Simulation study details

The simulations utilized haplotypes from the 1000 Genomes Phase 3 project[1]. In particular, we randomly selected a genetic region on Chromosome 22 with 1,000 consecutive common (minor allele frequency >5%) genetic variants. Based on the corresponding haplotypes, we generated genetic data for $n = 1000$ parents and, consequently, their offspring, assuming Hardy-Weinberg proportions and Mendel's laws of inheritance. Based on the simulated offspring genetic data, we generated quantitative traits using n_{causal} randomly selected genetic variants (with minor allele frequency >=10%), a pre-specified effect size β, and normally distributed random noise with unit variance. We considered additive and recessive genetic models for this simulation (assigning the specified effect to the minor allele). The simulations incorporated different configurations of $n_{causal} = 1,2$ and $\beta = 0.75, 1.0, 1.25, 2.5$ and simulated 100 independent replicates for each configuration (a small number of replicates did not contain genetic variation of the causal variants and were discarded, due to insufficient sample size for genetic variants with lower minor allele frequency). Based on the simulated data, we performed FBAT testing and applied the FBAT DAP-G fine-mapping implementation to the resulting summary statistics (z-scores and the estimated correlation matrix), evaluating the performance across various scenarios. The prior probabilities were chosen to be uniform across all genetic factors in the simulated region, and the fine-tuning parameter λ was set to one of the values $\lambda = 2.0, 1.3, 0.097$ to investigate the dependency of the results on these choices. As a comparison with an alternative method, we applied CAVIAR[2] to the vector of z-scores and the corresponding correlation matrix (-c 2 option, specifying a maximum of 2 causal genetic variants, default parameters otherwise).

AD APOE analysis details

Dataset

The application of FBAT DAP-G based on the AD data focused on the well-established AD risk loci Apolipoprotein E (APOE)[3]. The study data stems from the National Institute of Mental Health (NIMH) Genetics Initiative and was described in detail previously[4,5]. The quality-controlled dataset contained 1,393 individuals. In total, 431 nuclear families were available for the FBAT analysis. We extracted a +/- 500 kb window around the known APOE AD risk variant rs429358[6]. This genetic region contained 2716 common genetic variants (minor allele frequency >=1%). Most of the offspring were affected, but we incorporated unaffected family members by setting an offset parameter of 0.15.

APOE analysis using an additive model

Using an additive genetic model, FBAT DAP-G identified two signal clusters (Figure 10). The first signal cluster achieved a PIP of 99.7% and contained the four genetic variants rs429358, rs12721051, rs4420638, and rs56131196, aligned with expectations for the well-studied risk variant rs429358[6]. The strongest association in the second signal cluster was observed for rs11666285 ($p = 0.000233$, 74 informative families). This variant was previously reported to be associated with a family history of AD[7]. All genetic variants in the two signal clusters are reported in Table 1.

APOE analysis using a recessive model

The analysis based on a recessive model did not reveal additional insights. FBAT DAP-G identified two signal clusters, but these clusters mainly correspond to the first signal cluster of the additive model analysis (separated by allele) (Figure 11). Furthermore, the results indicate that the additive model provides a better description of the data.

Signal cluster	Variant	z-score	PIP
1	19:44908684:T:C	8.129	0.362
1	19:44918903:C:G	8.038	0.251
1	19:44919689:A:G	8.038	0.235
1	19:44919589:G:A	7.987	0.148
2	19:44998959:A:G	-3.680	0.053
2	19:44961706:C:T	-3.680	0.053
2	19:44989875:T:G	-3.680	0.053
2	19:45003514:T:A	-3.680	0.048
2	19:44973854:C:T	-3.531	0.040
2	19:45005766:C:A	-3.680	0.037
2	19:44993045:T:C	-3.680	0.037
2	19:44950506:A:G	-3.750	0.034
2	19:44947520:C:G	-3.665	0.032
2	19:44943921:C:T	-3.482	0.017
2	19:44938026:G:T	-3.396	0.012
2	19:44967300:C:G	-1.842	0.002
2	19:44948399:T:G	-1.882	0.001
2	19:44952331:T:C	-1.882	0.001
2	19:44949588:G:A	-1.882	0.001
2	19:44976194:A:T	-1.651	0.001
2	19:44947071:G:A	-1.869	0.001
2	19:45010596:C:T	-2.124	0.000

Table 1. All variants included in the two signal clusters identified by FBAT DAP-G, assuming an additive genetic model, in the Apolipoprotein E (APOE) locus for AD. PIP: posterior inclusion probability.

Run FBAT DAP-G using the FBAT tool

FBAT DAP-G can be applied by using the 'finemap' command. The command structure is the following:

finemap [-v filename] [variant-list]

Here, -v is an optional parameter to include more output information, while filename determines the names of the written files (with endings _R and _Z) of the computed correlation matrix and z-scores, respectively.

[variant-list] is a list of genetic variants in a region to which fine-mapping should be applied, separated by space. If no variant-list is provided, all genetic variants in the currently loaded dataset are considered.

One can modify the tuning parameters lambda (controls the addition of new candidate factors in the model) and the signal cluster correlation threshold (controls the definition of clusters based on within-correlations) with, for example:

log10_snp_thresh 2.0 (default value)

and

correlation_control_thresh 0.25 (default value)

The genetic model can be specified by

model a (default value, additive)

or

model r (recessive)

The files 'finemap_example.ped' and 'finemap_example.phe' can be used to test this approach. First:

./fbat

followed by:

load finemap_example.ped

load finemap_example.phe

trait y

finemap

quit

The top two results should be:

variant	incl_probability	cluster	log_BF	z-score
>>> m836_1	0.951	1	18.615	9.696
>>> m837_1	0.049	1	17.356	9.378

Here, the simulation underlying the example data assigned a true genetic effect to m836_1, while m837_1 is in strong LD (additive genetic model).

Supplementary Figures

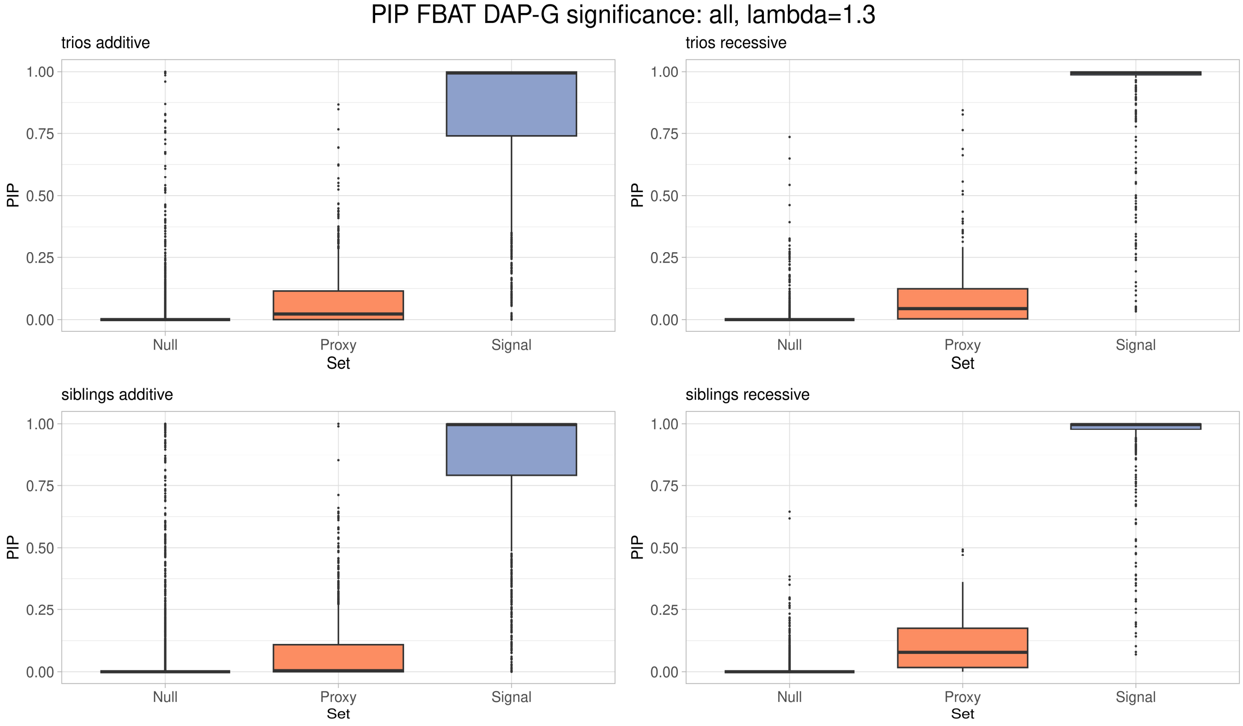

Figure 2. FBAT DAP-G posterior inclusion probabilities (PIPs) across all simulated replicates in which all true causal genetic effects achieved genome-wide significance ($p < 5 * 10^{-8}$). Results are shown stratified by study design and genetic model, leading to three different 'Sets'. 'Signal' denotes genetic association tests with true effect, 'Proxy' are genetic association tests in strong correlation ($r^2 \geq 0.8$) with causal genetic effects (LD in the case of an additive model). 'Null' denotes genetic association tests with no effect and not in correlation with signals. FBAT DAP-G parameter $\lambda = 1.3$. PIP: posterior inclusion probability.

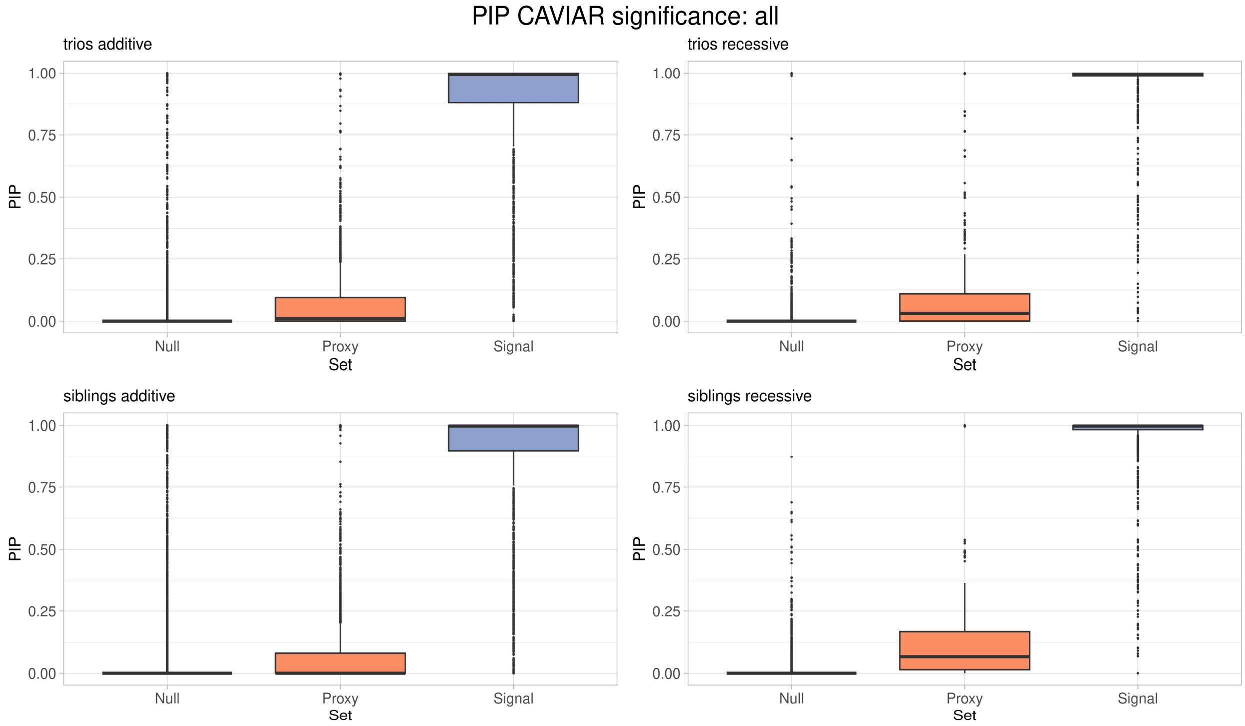

Figure 3. CAVIAR posterior inclusion probabilities (PIPs) across all simulated replicates in which all true causal genetic variants achieved genome-wide significance ($p < 5 * 10^{-8}$). Results are shown stratified by study design and genetic model, leading to three different 'Sets'. 'Signal' denotes genetic association tests with true effect, 'Proxy' are genetic association tests in strong correlation ($r^2 \geq 0.8$) with causal genetic effects (LD in the case of an additive model). 'Null' denotes genetic association tests with no effect and not in correlation with signals. PIP: posterior inclusion probability.

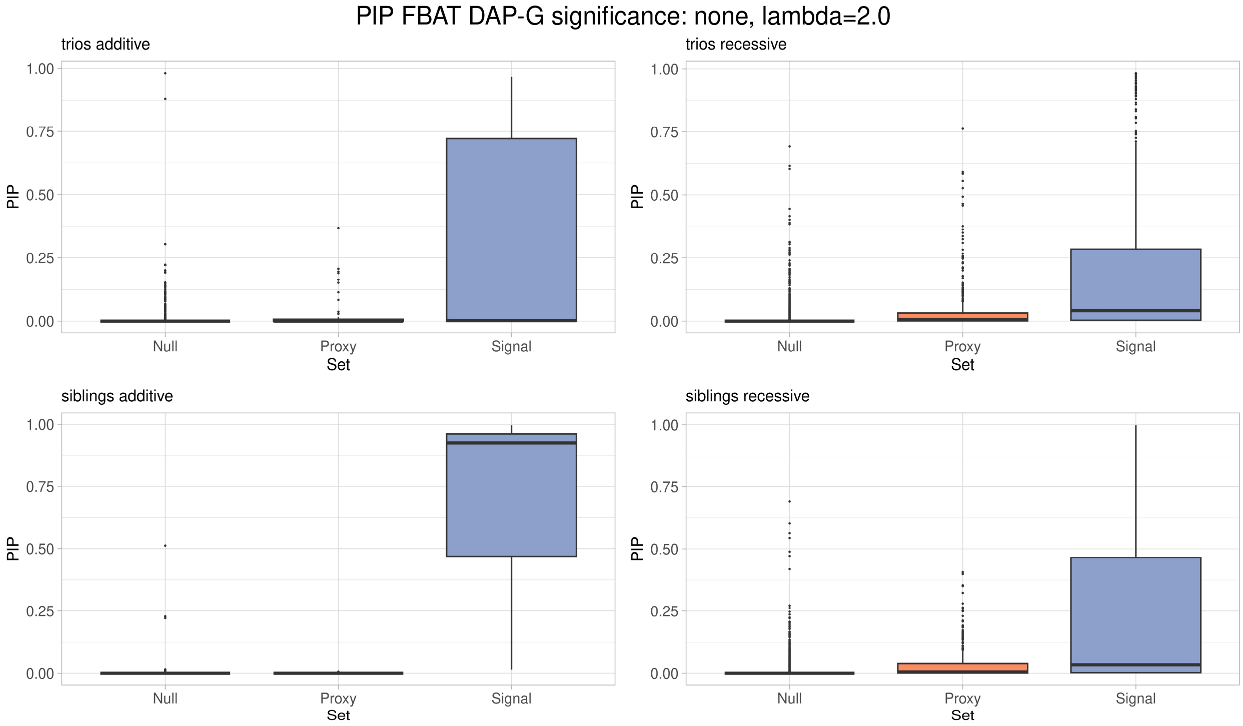

Figure 4. FBAT DAP-G posterior inclusion probabilities (PIPs) across all simulated replicates in which no true causal genetic effects achieved genome-wide significance ($p \geq 5 * 10^{-8}$). Results are shown stratified by study design and genetic model, leading to three different 'Sets'. 'Signal' denotes genetic association tests with true effect, 'Proxy' are genetic association tests in strong correlation ($r^2 \geq 0.8$) with causal genetic effects (LD in the case of an additive model). 'Null' denotes genetic association tests with no effect and not in correlation with signals. FBAT DAP-G parameter $\lambda = 2.0$. PIP: posterior inclusion probability.

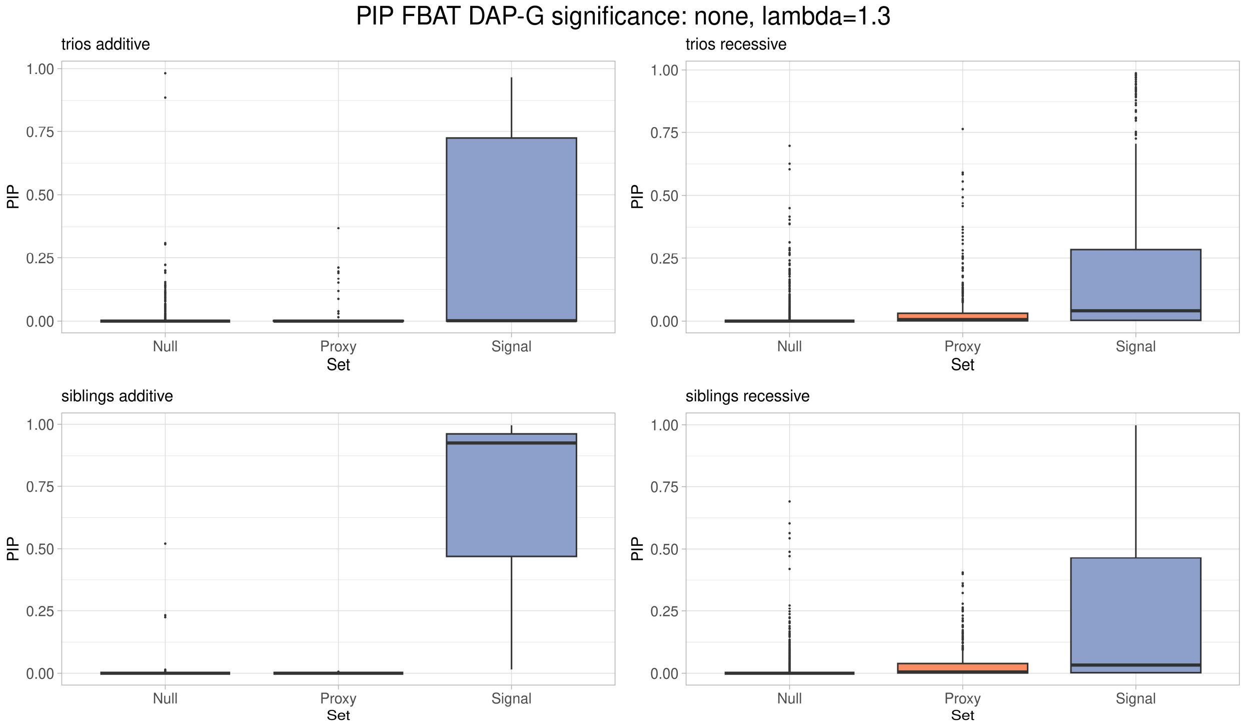

Figure 5. FBAT DAP-G posterior inclusion probabilities (PIPs) across all simulated replicates in which no true causal genetic effects achieved genome-wide significance ($p \geq 5 * 10^{-8}$). Results are shown stratified by study design and genetic model, leading to three different 'Sets'. 'Signal' denotes genetic association tests with true effect, 'Proxy' are genetic association tests in strong correlation ($r^2 \geq 0.8$) with causal genetic effects (LD in the case of an additive model). 'Null' denotes genetic association tests with no effect and not in correlation with signals. FBAT DAP-G parameter $\lambda = 1.3$. PIP: posterior inclusion probability.

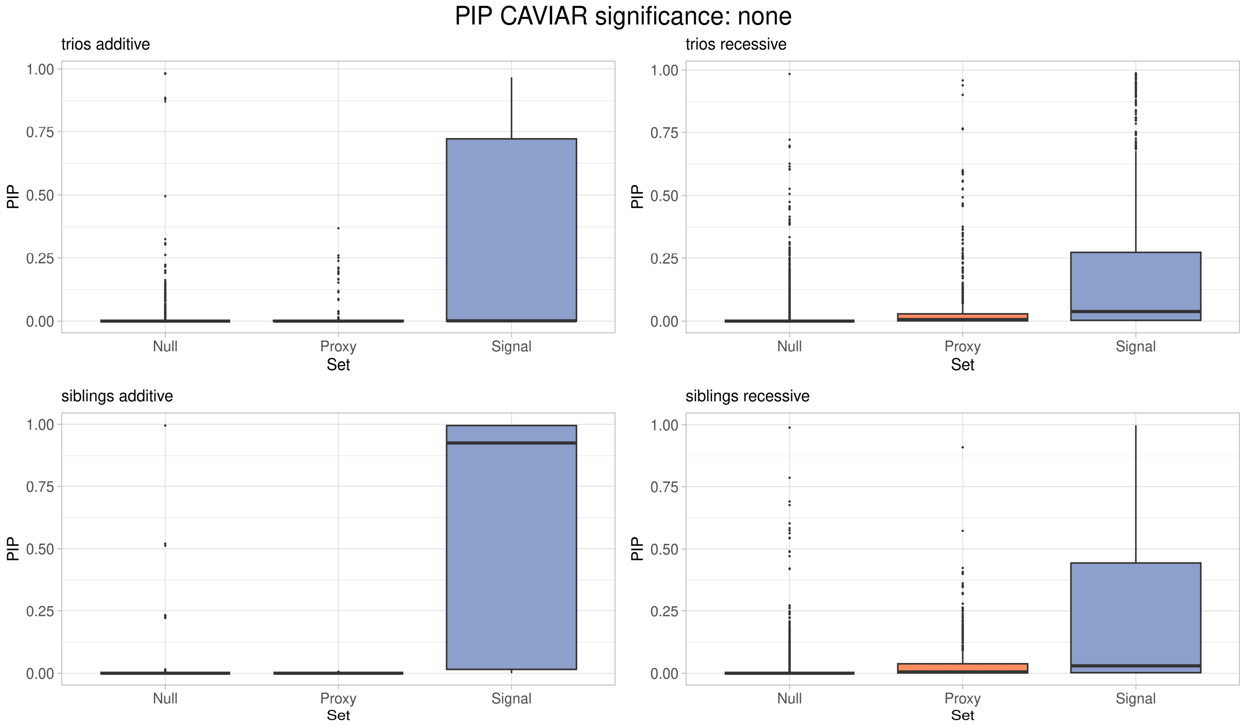

Figure 6. CAVIAR posterior inclusion probabilities (PIPs) across all simulated replicates in which no true causal genetic variants achieved genome-wide significance ($p \geq 5 * 10^{-8}$). Results are shown stratified by study design and genetic model, leading to three different 'Sets'. 'Signal' denotes genetic association tests with true effect, 'Proxy' are genetic association tests in strong correlation ($r^2 \geq 0.8$) with causal genetic effects (LD in the case of an additive model). 'Null' denotes genetic association tests with no effect and not in correlation with signals. PIP: posterior inclusion probability.

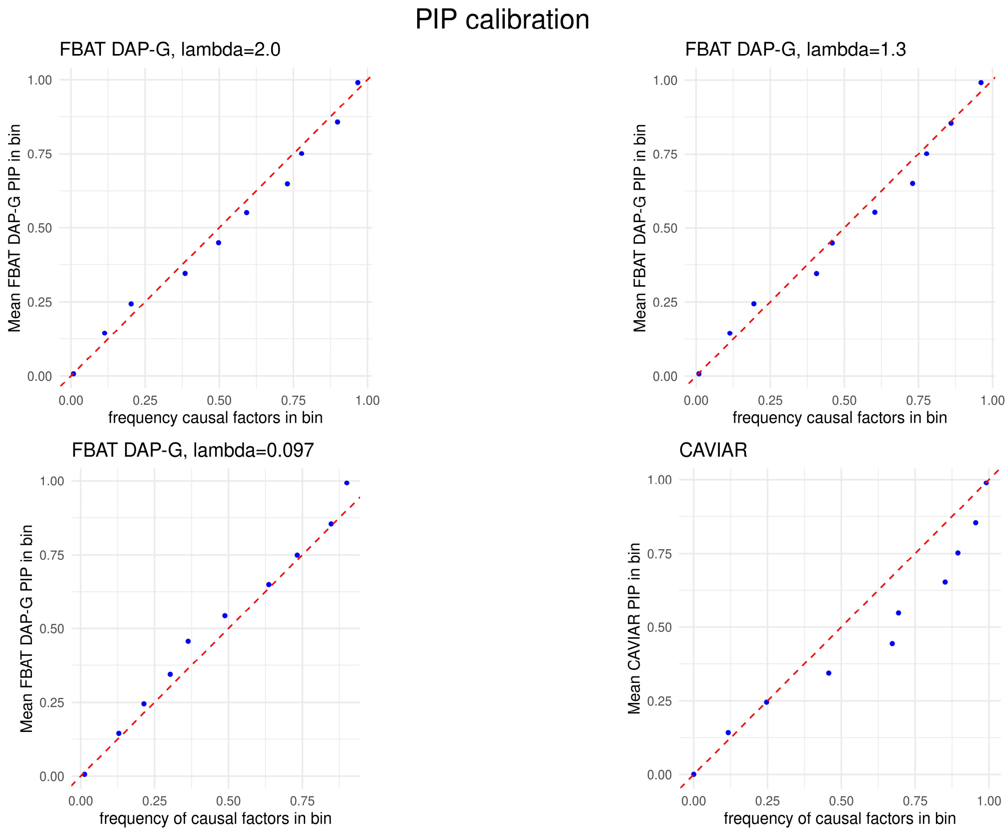

Figure 7. Frequency (true proportion) of causal genetic effects plotted against corresponding mean posterior inclusion probability (PIP) in 10 PIP bins (0%-10%, 10%-20%, 20%-30%, 30%-40%, 40%-50%, 50%-60%, 60%-70%, 70%-80%, 80%-90%, and 90%-100%), across all scenarios. The four plots correspond to FBAT DAP-G with $\lambda = 2.0, 1.3, 0.097$ and CAVIAR, respectively.

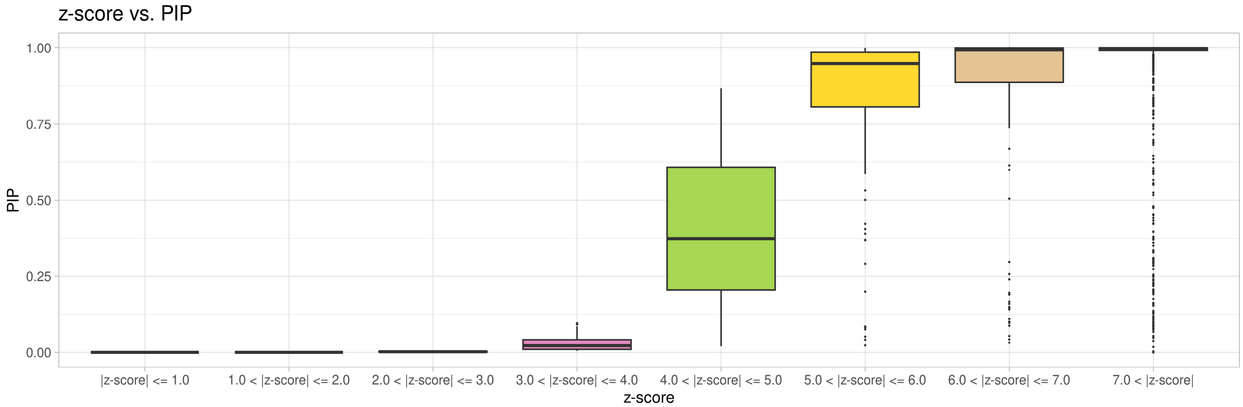

Figure 8. Relationship between the absolute value of the association z-score and the corresponding posterior inclusion probability (PIP) for the causal genetic effects in scenarios with exactly one causal variant, for FBAT DAP-G ($\lambda = 2.0$) and CAVIAR.

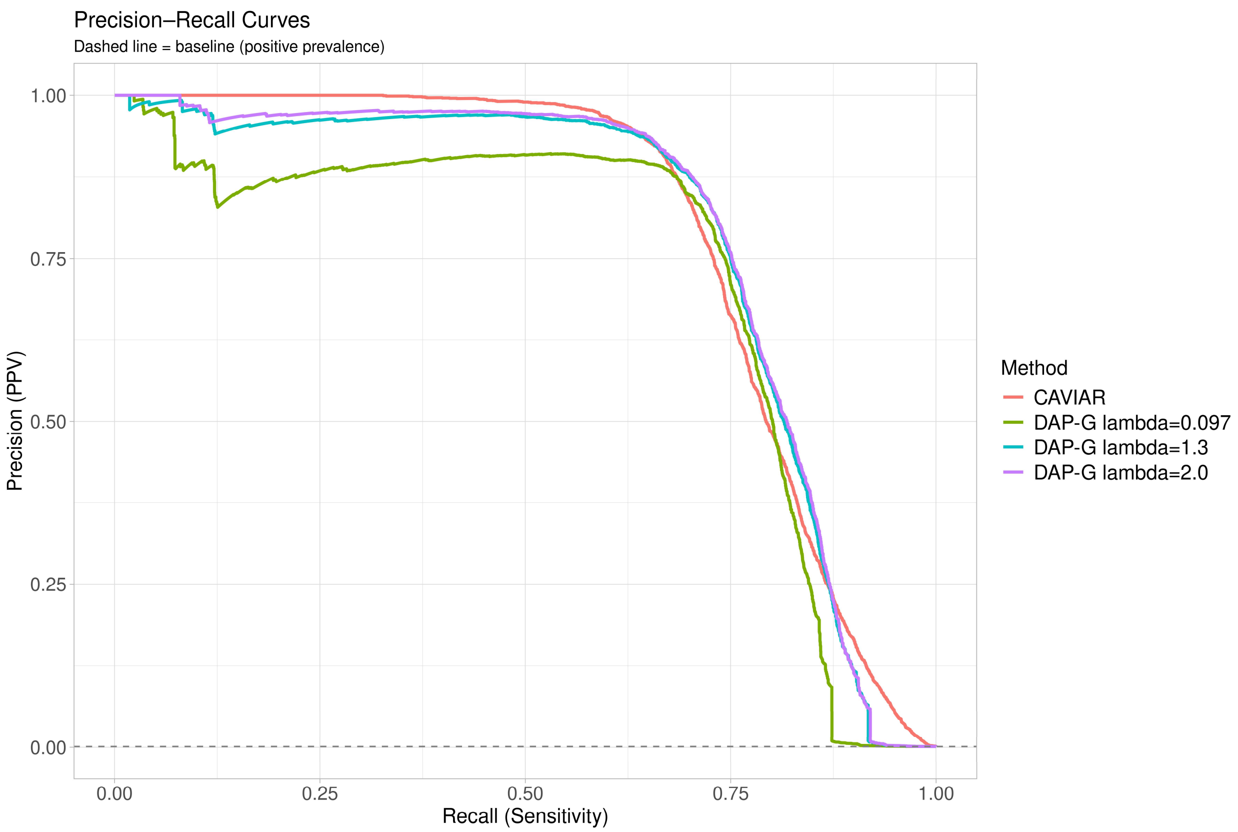

Figure 9. Precision-recall curves for DAP-G ($\lambda = 2.0, 1.3, 0.097$) and CAVIAR across all scenarios.

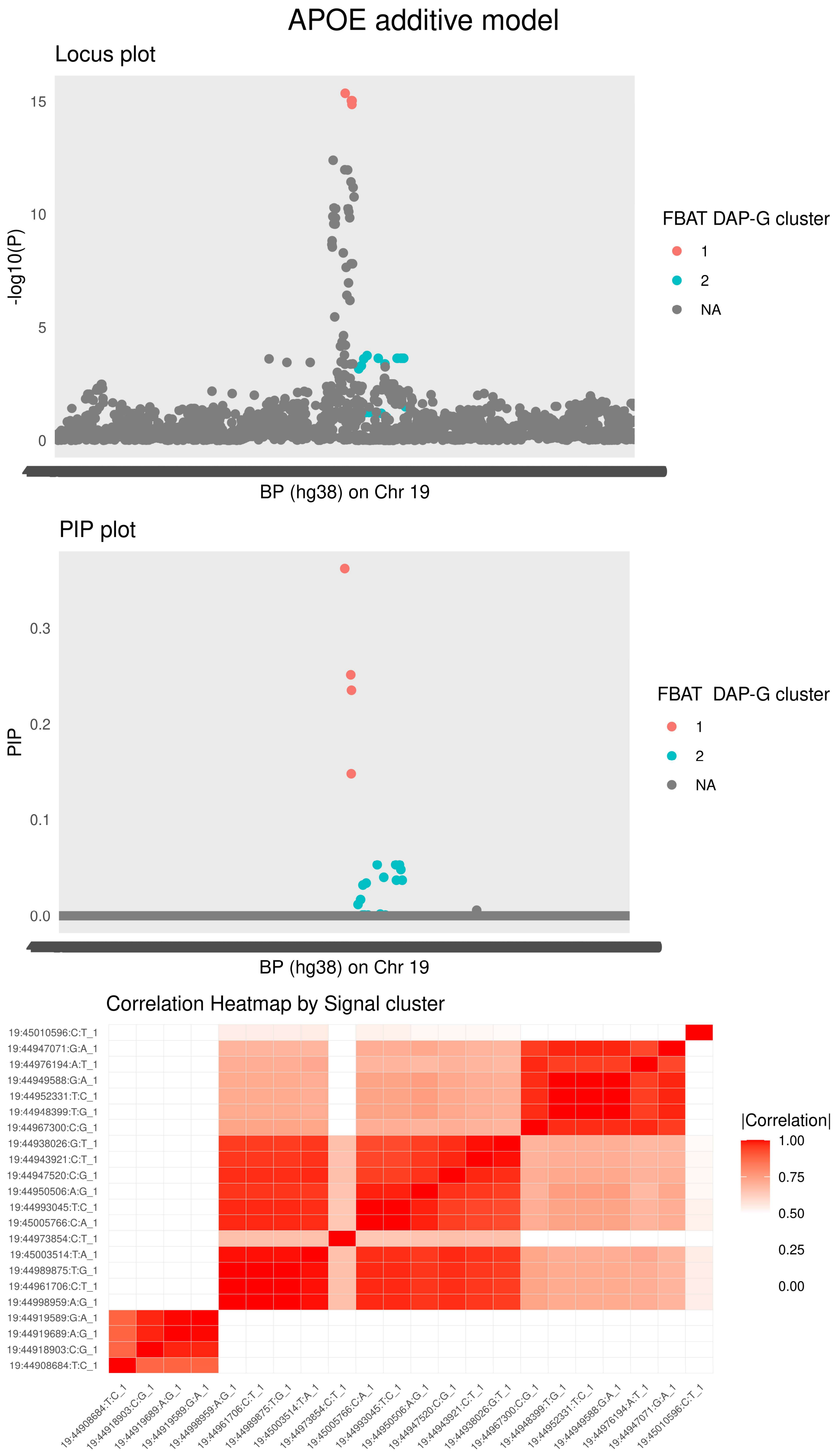
APOE additive model
Locus plot
-log10(P)
15
10
5
0
BP (hg38) on Chr 19
FBAT DAP-G cluster
1
2
NA
PIP plot
PIP
0.3
0.2
0.1
0.0
BP (hg38) on Chr 19
FBAT DAP-G cluster
1
2
NA
Correlation Heatmap by Signal cluster
19:45010596:C:T_1
19:44947071:G:A_1
19:44976194:A:T_1
19:44949588:G:A_1
19:44952331:T:C_1
19:44948399:T:G_1
19:44967300:C:G_1
19:44938026:G:T_1
19:44943921:C:T_1
19:44947520:C:G_1
19:44950506:A:G_1
19:44993045:T:C_1
19:45005766:C:A_1
19:44973854:C:T_1
19:45003514:T:A_1
19:44989875:T:G_1
19:44961706:C:T_1
19:44998959:A:G_1
19:44919589:G:A_1
19:44919689:A:G_1
19:44918903:C:G_1
19:44908684:T:C_1
|Correlation|
1.00
0.75
0.50
0.25
0.00

Figure 10. Regional association plot, posterior inclusion probability (PIP) information, and correlation/linkage disequilibrium (LD) heatmap for the identified signal clusters in the Apolipoprotein E (APOE) locus using FBAT DAP-G and an additive model. The LD heatmap plot visualizes absolute values of correlations between all test statistics assigned to the two signal clusters.

APOE recessive model

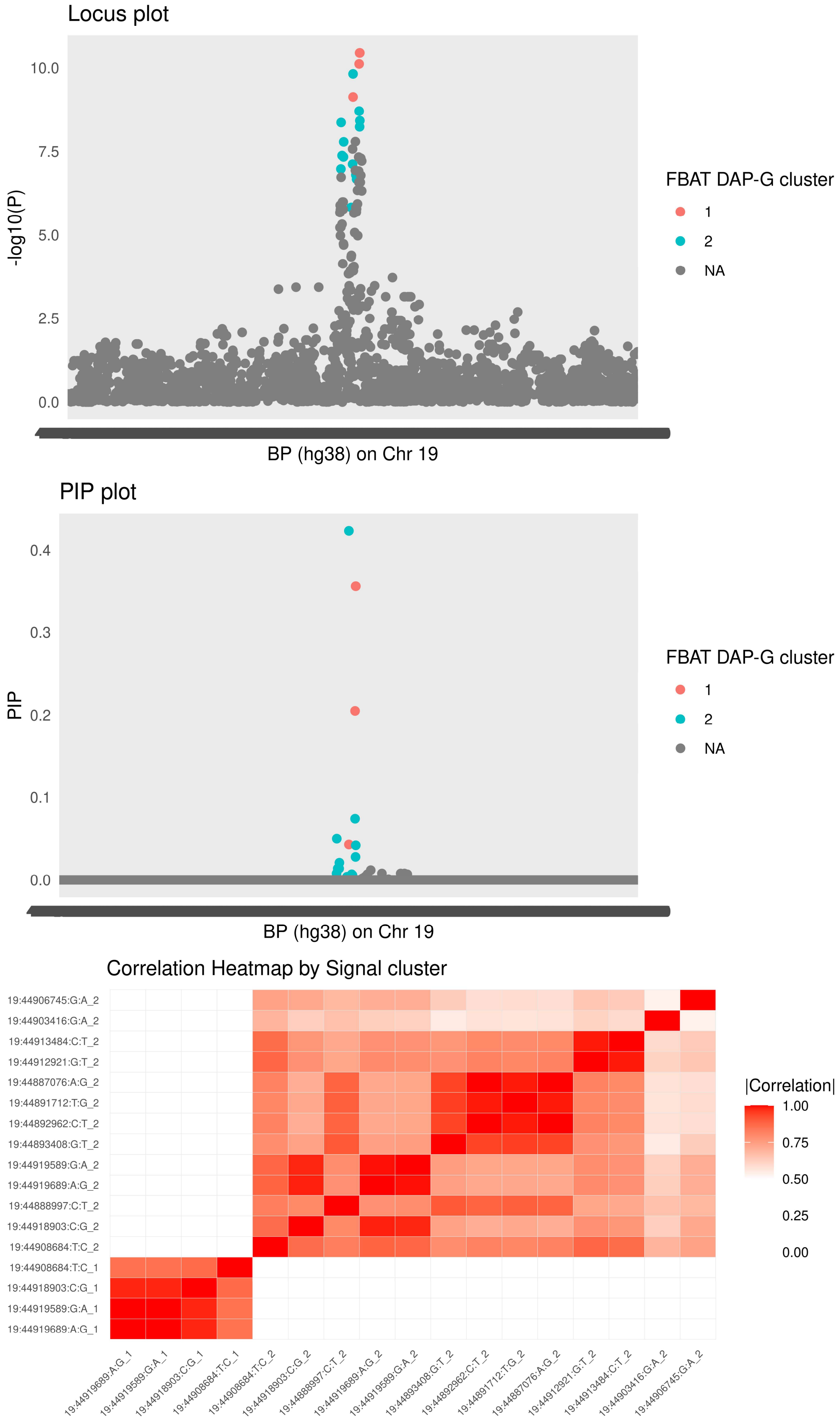

Figure 11. Regional association plot, posterior inclusion probability (PIP) information, and correlation heatmap for the identified signal clusters in the Apolipoprotein E (APOE) locus using FBAT DAP-G and a recessive model. The correlation heatmap plot visualizes absolute values of correlations between all test statistics assigned to the two signal clusters.

Runtime comparison DAP-G vs. CAVIAR

β	n_{causal}	Runtime DAP-G	Runtime CAVIAR
0.75	1	1.08 s	29.49 s
0.75	2	1.17 s	29.39 s
1.0	1	1.04 s	29.50 s
1.0	2	1.12 s	29.96 s
1.25	1	1.08 s	29.88 s
1.25	2	1.24 s	30.38 s
2.5	1	1.04 s	29.53 s
2.5	2	1.09 s	29.11 s

Table 2. Runtime comparison between the DAP-G approach and CAVIAR for different analysis scenarios (effect size and number of causal variants). For DAP-G, we selected $\lambda = 2.0$ and a maximum number of two causal variants for CAVIAR (-c 2 option). The runtime is based on an average across 10 runs. All scenarios were based on trios with an additive genetic model and 1000 genetic variants.

$n_{variants}$	Runtime DAP-G	Runtime CAVIAR
100	0.07 s	0.24 s
250	0.13 s	0.95 s
500	0.34 s	4.33 s
750	0.66 s	12.74 s
1000	1.07 s	29.46 s

Table 3. Runtime comparison between the DAP-G approach and CAVIAR for different numbers of genetic variants in the region of consideration. For DAP-G, we selected $\lambda = 2.0$ and a maximum number of two causal variants for CAVIAR (-c 2 option). The runtime is based on an average across 10 runs and considers the scenario with trios, an additive genetic model, and two causal genetic factors. For all analyses, the causal genetic factors were included in the selected set of genetic variants.

References

1. 1000 Genomes Project Consortium *et al.* An integrated map of genetic variation from 1,092 human genomes. *Nature* **491**, 56–65 (2012).
2. Hormozdiari, F., Kostem, E., Kang, E. Y., Pasaniuc, B. & Eskin, E. Identifying Causal Variants at Loci with Multiple Signals of Association. *Genetics* **198**, 497–508 (2014).
3. Fortea, J. *et al.* APOE4 homozygosity represents a distinct genetic form of Alzheimer's disease. *Nat. Med.* **30**, 1284–1291 (2024).
4. Prokopenko, D. *et al.* Whole-genome sequencing reveals new Alzheimer's disease–associated rare variants in loci related to synaptic function and neuronal development. *Alzheimers Dement.* **17**, 1509–1527 (2021).
5. Blacker, D. *et al.* ApoE-4 and age at onset of Alzheimer's disease: the NIMH genetics initiative. *Neurology* **48**, 139–147 (1997).
6. Kulminski, A. M. *et al.* Genetic and regulatory architecture of Alzheimer's disease in the APOE region. *Alzheimers Dement. Diagn. Assess. Dis. Monit.* **12**, e12008 (2020).
7. Schwartzentruber, J. *et al.* Genome-wide meta-analysis, fine-mapping and integrative prioritization implicate new Alzheimer's disease risk genes. *Nat. Genet.* **53**, 392–402 (2021).
